# Supplementary material for: Adrenal Insufficiency with Anticancer Tyrosine Kinase Inhibitors Targeting Vascular Endothelial Growth Factor Receptor: Analysis of the FDA Adverse Event Reporting System
Source: Cancers (Basel). 2022 Sep 22;14(19):4610. doi: 10.3390/cancers14194610 (PMC9559636; doi:10.3390/cancers14194610)
Supplement: Supplementary file 1 [file cancers-14-04610-s001.zip › cancers-1872373-supplementary.pdf]

**Table S1.** Affinity data (pKd/pIC50) from Chembl database.

| Drug | VEGFR 1 | VEGFR 2 | VEGFR 3 | PDGFR alpha | PDGFR beta | EGFR erbB1 | FGFR 1 | FGFR 2 | FGFR 3 | FGFR 4 | RET |
|---|---|---|---|---|---|---|---|---|---|---|---|
| **axitinib** | 10.00 | 9.72 | 10.00 | - | 6.39 | - | 6.57 | - | - | - | 6.98 |
| **cabozantinib** | 7.92 | 9.17 | 8.22 | - | - | 6.56 | 7.95 | - | - | - | 7.46 |
| **lenvatinib** | 7.66 | 8.54 | 8.28 | - | 6.50 | - | - | - | - | - | 7.75 |
| **nintedanib** | 7.76 | 8.02 | 8.14 | 8.68 | 7.97 | - | 6.33 | - | - | - | 8.52 |
| **pazopanib** | 7.61 | 7.61 | 7.41 | 8.31 | 7.32 | - | 6.38 | 6.68 | 6.17 | 5.55 | 6.07 |
| **regorafenib** | 7.89 | 8.38 | - | - | - | - | - | - | - | - | 6.90 |
| **sorafenib** | 7.51 | 7.55 | 7.41 | 6.78 | 6.98 | 7.64 | 6.58 | 5.57 | 5.30 | - | 7.69 |
| **sunitinib** | 8.74 | 7.42 | 7.30 | 7.92 | 8.51 | 5.55 | 5.84 | 6.24 | 6.19 | 5.68 | 7.88 |
| **tivozanib** | - | 6.47 | - | - | 5.62 | - | - | - | - | - | 7.92 |
| **vandetanib** | 6.58 | 7.10 | 5.96 | 6.64 | 7.06 | 7.37 | 5.77 | 5.96 | 5.47 | 5.64 | 6.78 |

**Table S2.** Disproportionality analysis. Reporting odd ratio (ROR) and information component (IC) with relevant 95% confidence/credibility interval (CI).

| Drug | | Vs all other drugs | | Vs other anticancer drugs | |
|---|---|---|---|---|---|
| | N | ROR (95%CI) | IC (95%CI) | ROR (95%CI) | IC (95%CI) |
| **bevacizumab** | 130 | 2.14 (1.79-2.55) | 1.08 (0.79-1.29) | 0.95 (0.79-1.14) | -0.07 (-0.36-0.14) |
| **ranibizumab** | 0 | - | - | - | - |
| **ramucirumab** | 8 | 2.55 (1.09-5.03) | 1.22 (0-2.02) | 1.14 (0.49-2.26) | 0.18 (-1.04-0.98) |
| **aflibercept** | 2 | - | -2.56 (-5.15--1.17) | - | -3.68 (-6.28--2.29) |
| **lenvatinib** | 67 | 5.86 (4.54-7.46) | 2.48 (2.08-2.77) | 2.65 (2.04-3.38) | 1.36 (0.96-1.65) |
| **sorafenib** | 22 | 1.40 (0.87-2.12) | 0.47 (-0.24-0.97) | 0.62 (0.39-0.95) | -0.66 (-1.37--0.16) |
| **vandetanib** | 1 | - | -0.13 (-3.92-1.56) | - | -1.02 (-4.8-0.67) |
| **cabozantinib** | 74 | 4.09 (3.21-5.15) | 1.99 (1.6-2.27) | 1.84 (1.44-2.32) | 0.86 (0.47-1.13) |
| **sunitinib** | 62 | 2.16 (1.65-2.78) | 1.09 (0.67-1.39) | 0.96 (0.74-1.24) | -0.05 (-0.47-0.25) |
| **regorafenib** | 1 | - | -2.29 (-6.07--0.6) | - | -3.39 (-7.17--1.7) |
| **axitinib** | 58 | 6.32 (4.79-8.19) | 2.58 (2.14-2.89) | 2.85 (2.16-3.7) | 1.47 (1.03-1.78) |
| **pazopanib** | 26 | 1.56 (1.02-2.29) | 0.63 (-0.03-1.09) | 0.70 (0.45-1.02) | -0.5 (-1.16--0.04) |
| **tivozanib** | 0 | - | - | - | - |
| **nintedanib** | 10 | 0.84 (0.4-1.54) | -0.24 (-1.32-0.48) | 0.37 (0.18-0.69) | -1.36 (-2.44--0.64) |
| **VEGFR mAbs** | 130 | 1.72 (1.43-2.05) | 0.77 (0.48-0.98) | 0.94 (0.78-1.12) | -0.08 (-0.37-0.13) |
| **VEGFR TKIs** | 314 | 2.71 (2.42-3.04) | 1.40 (1.22-1.54) | 1.21 (1.07-1.36) | 0.25 (0.07-0.39) |
| **VEGFR TKIs (monotherapy)** | 180 | 2.00 (1.71-2.32) | 0.97 (0.73-1.15) | 1.52 (1.30-1.78) | 0.55 (0.30-0.72) |
| **VEGFR Inhibitors** | 453 | 2.22 (2.02-2.44) | 1.11 (0.95-1.22) | 1.05 (0.95-1.16) | 0.06 (-0.1-0.17) |
| **CDK4/6 Inhibitors** | 5 | 0.08 (0.02-0.2) | -3.37 (-4.94--2.39) | 0.03 (0.01-0.08) | -4.52 (-6.08--3.54) |
| **nivolumab and ipilimumab** | 1161 | 26.36 (24.75-28.05) | 4.50 (4.40-4.57) | 15.4 (14.32-16.54) | 3.35 (3.26-3.42) |
| **JAK Inhibitors** | 107 | 0.8 (0.65-0.96) | -0.32 (-0.64--0.09) | 0.49 (0.38-0.62) | -0.98 (-1.38--0.69) |

**Table S3.** Disproportionality analysis (ROR with 95%CI) adjusted for concomitant glucocorticoid and immunotherapy.

| VEGFR-TKI | ROR (95%CI) vs other anticancer drugs |
|---|---|
| **axitinib** | 1.61 (1.23-2.08) |
| **cabozantinib** | 1.41 (1.10-1.87) |
| **lenvatinib** | 1.10 (0.85-1.38) |
| **sunitinib** | 2.16 (1.66-2.76) |
| **pazopanib** | 1.40 (0.93-2.02) |
